# High Fischer Ratio Oligopeptides of Gluten Alleviate Alcohol-Induced Liver Damage by Regulating Lipid Metabolism and Oxidative Stress in Rats

**DOI:** 10.3390/foods13030436

**Published:** 2024-01-29

**Authors:** Penghui Zhao, Yinchen Hou, Xinyang Chen, Mingyi Zhang, Zheyuan Hu, Lishui Chen, Jihong Huang

**Affiliations:** 1 Food Laboratory of Zhong Yuan, Luohe 462300, China; 2 School of Biological Engineering, Henan University of Technology, Zhengzhou 450001, China; 3 College of Food and Biological Engineering, Henan University of Animal Husbandry and Economy, Zhengzhou 450044, China; 4 Collaborative Innovation Center of Functional Food Green Manufacturing, Xuchang 461000, China; 5 State Key Laboratory of Crop Stress Adaptation and Improvement, College of Agriculture, Henan University, Kaifeng 475004, China

**Keywords:** wheat gluten, high Fischer ratio oligopeptides, oxidative stress, liver protection

## Abstract

High Fischer ratio oligopeptides (HFOs) exhibit diverse biological activities, including anti-inflammatory and antioxidant properties. HFOs from gluten origin were prepared through fermentation and enzymatic hydrolysis and then characterized using free amino acid analysis and scanning electron microscopy (SEM). Following intervention, the levels of serum total cholesterol (TC), triglyceride (TG), alanine aminotransferase (ALT), aspartate aminotransferase (AST), and hepatic malondialdehyde (MDA) in the rats significantly decreased (*p* < 0.05). Simultaneously, there was an increasing trend in superoxide dismutase (SOD) levels, and glutathione (GSH) levels were significantly elevated (*p* < 0.05). The mRNA expression levels of alcohol metabolism-related genes (ADH4, ALDH2, and CYP2E1) exhibited a significant increase (*p* < 0.05). Histological examination revealed a reduction in liver damage. The findings indicate that high Fischer ratio oligopeptides, prepared through enzymatic and fermentation methods, significantly improve lipid levels, ameliorate lipid metabolism disorders, and mitigate oxidative stress, and exhibit a discernible alleviating effect on alcoholic liver injury in rats.

## 1. Introduction

The liver stands as one of the body’s five major organs, central to numerous metabolic activities [1]. The escalating concern over alcohol-related liver damage (ALD), stemming from excessive alcohol consumption, highlights the increasing human mortality rate attributed to ALD [2]. The primary clinical presentations encompass cirrhosis [3], liver fibrosis [4], and steatohepatitis [5]. In more severe instances, these conditions may progress to cancer [6,7]. Notably, 4.1% of global new cancer cases in 2020 were attributed to alcohol consumption [8]. ALD exhibits associations with specific cardiovascular conditions, as indicated by research [9]. The pathways instigating liver injury in alcoholic liver damage predominantly involve oxidative stress and the pivotal role of proinflammatory cytokines [10,11]. A previous study showed that the impact of ethanol on cAMP signaling induces a dysregulated inflammatory response and modifies lipid metabolism, and they confirmed a substantial upregulation in the expression of cAMP-degrading phosphodiesterase 4 (PDE4) subfamily enzymes, leading to markedly reduced cAMP levels in severe ALD patients’ liver tissue [12]. 

In mitigating oxidative stress, carrot Polysaccharide-I (CPS-I), administered at 300 mg/kg/day, exhibited promising effects by elevating ethanol dehydrogenase (ADH) and acetaldehyde dehydrogenase (ALDH) levels. This intervention ameliorated liver pathologies typified by lipid accumulation and reduced the number of lipid droplets [13]. In the context of an aging and liver injury model, it was observed that the consumption of alcohol led to an elevation in both neutrophil SIRT1 and serum miR-223 levels as a result of inflammatory reactions [14]. However, the aging group had increased inflammatory mediator and reactive oxygen species (ROS) levels due to the decreases in SIRT1 and miR223, which exacerbated liver injury. The results showed that neutrophil SIRT1, miR-223, and serum miR-224 levels were negatively correlated with age, and the deletion of the SIRT1 gene in neutrophils from the aging group significantly enhanced alcohol-induced liver injury and inflammation. This provides a new target for the treatment of ALD.

HFO is a protein precursor or hydrolysate consisting of 2–9 amino acids with a ratio of the amount of branched-chain amino acids (BCAAs), including leucine (Leu), isoleucine (Ile), and valine (Val), to the amount of aromatic amino acids (AAAs), including tyrosine (Tyr), phenylalanine (Phe), and tryptophan (Trp), of substance F greater than 20. The protein precursor or hydrolysate is composed of 2–9 amino acids. It has a unique amino acid composition, physiological regulatory function, and easy digestion and absorption properties. It has great potential in the clinical treatment of liver disease and has attracted increased amounts of attention [15]. Studies in the relevant literature have documented the ability of HFO to slow lipid oxidation and oxidative stress.

HFO was derived from Antarctic krill using a two-step enzymatic digestion method, and was shown to scavenge free radicals and inhibit lipid peroxidation [16]. The induction of oxidative stress resulting from hepatic mitochondrial damage is recognized as a significant factor in the pathogenesis of hepatocellular carcinoma (HCC). BCAAs have demonstrated the potential to alleviate oxidative stress in individuals with iron overload by enhancing mitochondrial function and modulating iron metabolism through the upregulation of hepcidin-25 levels, the hepatitis C virus polyprotein HCVTgM, or hepatitis C HCV-associated advanced fibrosis, thereby slowing liver injury [17]. Tamai investigated the effects of BCAA treatment on cirrhosis and hepatic steatosis using a cirrhosis model. These findings indicated that BCAAs reduced hepatocyte injury by decreasing lipid peroxidation and maintaining mitochondrial integrity in cirrhotic livers [18]. Furthermore, BCAA administration was associated with a reduction in hepatic steatosis, as evidenced by decreased liver and serum TG levels. BCAAs also improved lipid distribution and decreased fat accumulation in the liver, exerting a beneficial effect on hepatic steatosis. Importantly, BCAAs exhibited superior anti-lipid peroxidation activity compared to L-carnitine. Metadoxine accelerates the excretion of ethanol and its metabolites, acetaldehyde and ketone bodies, via the kidneys, thus speeding up the restoration of liver function, protecting liver cell membranes, and preventing liver toxicity caused by excessive alcohol intake [19]. Therefore, this study used metadoxine as a positive drug to observe the effect of HFO in a controlled manner.

In recent years, numerous investigations have been conducted to explore the preparation techniques and corresponding characterization of HFO. However, the body of research pertaining to the efficacy of HFO in protecting the liver is relatively rare. The precise role of HFO in the mechanisms underlying liver protection requires further examination. In this particular investigation, an ALD model was established in rats through the administration of alcohol via gavage. Liver and serum-related physiological and biochemical indicators were measured, while the expression of relevant inflammatory factors and alcohol metabolizing enzymes was monitored using quantitative polymerase chain reaction (qPCR). The potential mechanism underlying the hepatoprotective efficacy of HFO in rats experiencing alcoholic liver injury was subsequently investigated. This study provides a theoretical foundation for the future development and utilization of HFO in the realms of food and pharmaceuticals.

## 2. Materials and Methods

### 2.1. Chemicals and Reagents

Wheat gluten (WG) was purchased from Thehenan Feitian Agriculture Co., Ltd. (Zhengzhou, China), *Bacillus subtilis* (B53) served as a preservation strain in laboratory settings at the Henan Province Industrial Microbial Species Preservation Center (Zhengzhou, China). Total RNA extraction and HiScript^®^II Q RT SuperMix kits were purchased from Vazyme Biotechnology Co., Ltd. (Nanjing, China). Main reagents: Tetraethoxypropane, NBT, MET from Shanghai Maclean Biochemical Co., Ltd. (Shanghai, China) and DTNB, SDS, and EDTA from Beijing Sorabio Technology Co., Ltd. (Beijing, China). DPPH, ABTS, TCA, FeSO4, and H_2_O_2_ reagents utilized in the experiments were procured from Shanghai Yuanye Biotechnology Co., Ltd. (Shanghai, China). All the chemicals used in this study were of analytical grade.

### 2.2. Preparation of HFO

Enzymatic high Fischer ratio oligopeptides (EHFOs) were prepared following the method outlined by Yan et al. [20]. Alkaline protease (≥200 U/mg) and flavorzyme (≥120 U/mg) were selected for stepwise hydrolysis. The hydrolysis conditions for alkaline protease included an enzyme dosage of 8000 U/g, pH 10, material-liquid ratio of 1/10 (*w*/*v*), enzyme digestion temperature of 55 °C, and enzyme digestion time of 3 h, achieving a hydrolysis degree of 32.74%. The two-step enzymatic conditions for flavorzyme involved a pH of 7, enzymatic temperature of 55 °C, enzyme addition of 3200 U/mL, and an enzymatic time of 3 h. After hydrolysis by flavorzyme, the supernatant was extracted by centrifugation at 4 °C at 6000× *g*.

Fermented high Fischer ratio oligopeptides (FHFOs) were produced using *Bacillus subtilis* as the fermentation strain. This involved mixing gluten powder with sterile water at a ratio of 1/9 (*w*/*v*), sterilizing at 121 °C for 30 min with an inoculum amount of 3%, and fermenting at 37 °C for 120 h, followed by centrifugation at 6000× *g* at 4 °C. The resulting supernatant was extracted and set aside. The above enzymatic and fermentation hydrolysates were subjected to high Fischer ratios by the method mentioned [21], with mentioned modifications. The supernatant from the initial two steps was acidified to pH 3, and powdered activated carbon was introduced at a ratio of 1/15 (*w*/*v*) to facilitate the adsorption of aromatic amino acids for 3 h. Following adsorption, the supernatant was retrieved through centrifugation at 10,000× *g* and subsequently filtered through a 0.10 μm microporous membrane before freeze-drying to yield EHFO and FHFO, respectively.

### 2.3. Characterization of HFO

#### 2.3.1. Amino Acid Analysis

The amino acid composition of the samples was determined utilizing an automated amino acid analyzer (Sykam, Eresing, Germany) in accordance with the procedures outlined in the pertinent literature [20]. In particular, 0.1 g of the sample is precisely measured and placed into a hydrolysis tube. Subsequently, 10 mL of concentrated sulfuric acid with a concentration of 6 mol/L is meticulously added in a fume hood environment. The tube was then evacuated and filled with nitrogen, and this process was repeated three times. The screw cap was tightened under nitrogen and the hydrolysis was carried out for 22 h at a constant temperature of 110 °C in an oven. Upon reaching room temperature, the hydrolysis solution was filtered into a 50 mL volumetric flask and the volume was adjusted with ultrapure water. Next, 1.0 mL of the hydrolysate was accurately aspirated into a glass test tube, dried under reduced pressure at 70 °C, and redissolved with 2.0 mL of 0.02 mol/L hydrochloric acid solution. The solution was vortexed, passed through a 0.22 m aqueous filter membrane, and then measured using an automated analyzer. The Fischer ratio of the HFO can be determined using the formula: Fischer ratio = BCAA content/AAA content.

#### 2.3.2. Microscopic Morphology (SEM)

The morphologies of WG, EHFO, and FHFO were analyzed using a Zeiss EVLS-15 scanning electron microscope [22]. The microscope was operated at an acceleration voltage of 5 kV and an observation multiple of 1000 times. Prior to observation, a thin layer of 15 nm gold foil was evenly deposited on the surface of the sample using an ion ejector.

#### 2.3.3. Determination of Antioxidant Activity

(1) DPPH free radical scavenging

The DPPH free radical scavenging rate was determined with reference to the literature [23]. Specifically, 2.0 mL of HFO solution with different concentrations (1, 2, 3, 4, and 5 mg/mL) were taken separately, and 2.5 mL of 100 mol/L DPPH methanol solution was added. The reaction was carried out for 30 min at room temperature, protected from light, and deionized water was used as the zeroing solution.

(2) OH radical scavenging rate experiment

The OH radical scavenging rate was determined according to the literature [23], so 1.0 mL of HFO solution with different concentrations (1, 2, 3, 4, and 5 mg/mL) was taken separately, and 1.0 mL of 9 mmol/L FeSO_4_, 1.0 mL of 9 mmol/L salicylic acid, and 0.5 mL of 0.1% H_2_O_2_ were added sequentially. The reaction was agitated in a water bath at 37 °C for a duration of 30 min. Deionized water was used as the zeroing solution.

(3) Measurement of ABTS free radical scavenging capacity

The ABTS free radical scavenging capacity was determined with reference to the literature [23]. Different concentrations (1, 2, 3, 4, and 5 mg/mL) of HFO solution in 0.2 mL were separately obtained. To initiate the reaction, 4 mL of ABTS radical reserve solution was added and thoroughly mixed. The resulting mixture was then incubated for 6 min at room temperature while shielded from light. Deionized water was used as the zeroing solution.

(4) Measurement of iron reducing power

The iron reducing power was determined according to the literature [24]. A 1.0 mL aliquot of HFO solution with varying concentrations (1, 2, 3, 4, 5 mg/mL) was pipetted, followed by the addition of phosphate buffered saline (PBS) and K_3_Fe(CN)_6_. After thorough mixing, the solution was placed in a water bath at 50 °C for a constant reaction time of 20 min. Subsequently, 1.0 mL of 10% trichloroacetic acid (TCA) solution was added. From this mixture, 2.5 mL was extracted and combined with 2.5 mL of deionized water, 1.0 mL of FeCl_3_ solution with a mass concentration of 0.1%, and 1.0 mL of FeCl_3_ solution with deionized water for zero adjustment. The optical density (OD) at 700 nm was measured to determine the reducing power, using deionized water as the zeroing solution.

### 2.4. Animal Experimental Design

Male rats weighing approximately 180 g and specific pathogen-free (SPF) grade were sourced from Huaxing Laboratory Animal Co., Ltd. (Henan, China) (SCXK (Yu) 2019-0002). The study followed ethical guidelines approved by the Institutional Animal Care and Use Committee of Henan University of Technology (protocol code: P20220328-1). Rats were housed in a temperature-controlled room (24 ± 1 °C, 50 ± 5% humidity, 12-h light-dark cycle) with ad libitum access to standard chow and water for acclimatization. After one week, the rats were randomly divided into five groups (*n* = 10): the blank (NC), model (AC), positive control (PC, metadoxine, 50 mg/kg body weight), E-HFO (100 mg/kg body weight), and F-HFO (100 mg/kg body weight) groups. Following 15 days, all groups, except for the NC group, received twice-daily gavages of 56% ethanol (5 mL/kg body weight). After the final administration, the rats underwent a 12-h fasting period. Rats were anesthetized with isoflurane, blood samples were collected from the eyes for serum, and livers were weighed, bagged, and stored at −80 °C.

### 2.5. Liver Index Assay

The liver tissue and body weight of the rats before dissection were recorded, and the liver index was calculated.

(1) $$Liver\;index=\frac{Liver\;weight(g)}{rat’s\;last\;body\;weight(g)}\times 100\%$$

### 2.6. Serum Biochemical Analysis

Blood samples stored at room temperature for 30 min were centrifuged at 3500× *g* at 4 °C and then rested for 20 min to collect the serum [25]. Total cholesterol (TC), triacylglycerol (TG), low-density lipoprotein cholesterol (LDL-C), high-density lipoprotein cholesterol (HDL-C), aspartate aminotransferase (AST), and alanine aminotransferase (ALT) levels were measured by the PUZS-300 automatic biochemical analyzer (Plan Medical, Beijing, China). 

### 2.7. Antioxidant Analysis of the Liver

Liver tissue was taken and homogenized by adding nine times the volume of pre-cooled saline. After centrifugation for 15 min, the supernatant was collected. The levels of superoxide dismutase (SOD), reduced glutathione (GSH), and malondialdehyde (MDA) were determined by referring to the method reported by Yang et al. [26]. The test was repeated three times for each sample.

### 2.8. Histological Analysis

Fresh liver tissues were preserved by immersing them in a 4% solution of paraformaldehyde for 24 h. Subsequently, they were subjected to a series of treatments involving saline, ethanol, and xylene. The tissues were then embedded in conventional paraffin to obtain sections approximately 5 μm in thickness [27]. These sections were stained with hematoxylin and eosin (H and E), and then analyzed for morphological alterations using Upright optical microscope (Nikon Eclipse E100, Nikon, Minato City, Japan).

### 2.9. RNA Extraction and mRNA Quantification by qRT-PCR

The changes in the expression of alcohol metabolizing enzymes were quantified through qRT-PCR. Total RNA was extracted from the rat liver samples in strict accordance with the kit instructions and reverse transcribed to obtain the reverse transcription product cDNA. PCR reaction was performed using ChamQ Universal SYBR qPCR Master Mix, with GAPDH serving as the internal reference gene. The PCR amplification program was set as follows: stage 1, predenaturation at 95 °C for 3 min; and stage 2, denaturation at 95 °C for 3 s. The reaction was annealed and extended at 60 °C for 30 s for 40 cycles. The relative quantification of gene expression was determined using the 2^−ΔΔCT^ method. The specific primers utilized for the qRT-PCR are provided in Table 1.

### 2.10. Statistical Analysis

All the experimental data are expressed as mean ± SD. ANOVA was analyzed using SPSS 20.0 software. Duncan’s multivariate range test was used to determine the mean difference and a significance level of *p* < 0.05 was employed to determine statistical significance in the study. Additionally, Origin 2018 software was utilized for creating charts or graphs as part of the data visualization process.

## 3. Results

### 3.1. Amino Acid Analysis

The amino acid compositions of EHFO and FHFO are shown in Table 2. The BCAA content of EHFO was 6.472 mg/mL, and the AAA content was only 0.276 mg/mL. BCAAs accounted for 22.092% of the total amino acids, whereas AAAs represented only 0.943%, and the Fischer ratio was 23.436 (Table 3). Similarly, the BCAA content of FHFO was 6.843 mg/mL, and the AAA content was only 0.272 mg/mL. BCAAs accounted for 27.721% of the total amino acids, while AAAs represented only 1.100%, yielding a Fischer ratio of 25.195. Therefore, the hydrolysate obtained by enzymatic hydrolysis and fermentation of gluten under optimal conditions meets the requirement of a high Fischer ratio after adsorption by activated carbon. It maximizes the retention of other essential amino acids, excluding AAAs, during the adsorption process. It can be reasonably inferred that the EHFO and FHFO prepared here are hydrolysis products endowed with diverse physiological functions and nutritional value.

### 3.2. Micromorphology of High Fischer Ratio Oligopeptides

**Figure 1:**
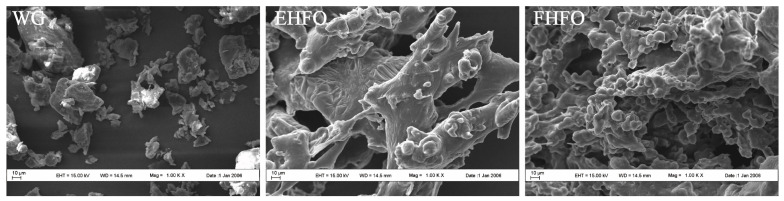
Scanning electron microscopy images of WG, EHFO, FHFO.

The SEM images depicted in Figure 1 reveal distinct variations in the microstructures of the WG, EHFO, and FHFO samples. In the absence of enzymatic and fermentation treatment, the gluten surface appears loose, lacking internal linkages to form a reticulated structure, and exhibiting numerous irregular block structures. Conversely, EHFO and particularly FHFO exhibit a transition from loose surfaces to the development of a cohesive three-dimensional network structure. This implies the gradual degradation of gluten subsequent to enzyme-catalysis and fermentation, resulting in molecular stretching that exposes the internal hydrophobic region, thereby inducing partial depolymerization of the structure. The emergence of compact cavities within the microstructure of FHFO was accompanied by surface linkage initiation. These observations suggest that alterations in the microstructure might stem from protein decomposition triggered by proteases generated during *Bacillus subtilis* fermentation and the erosive impact of microorganisms. The abundance of microscopic holes further corroborated the experimental findings of protein degradation, resulting in the release of numerous free amino acids, as previously observed. Microstructural analysis indicates that during the fermentation process, FHFO undergoes gradual degradation through the collaborative action of endogenous proteases and microbial-secreted proteases, yielding smaller protein and peptide molecules, thereby stretching and exposing the molecular structure.

### 3.3. Antioxidant Analysis of HFO

**Figure 2:**
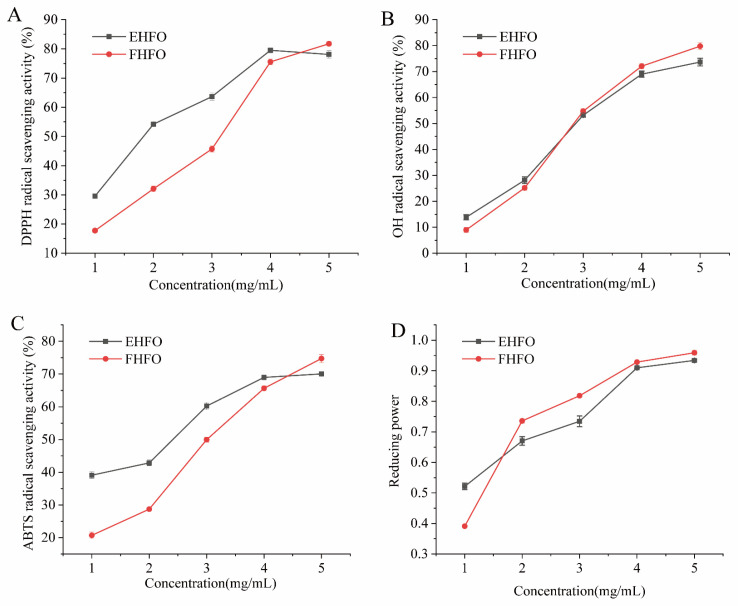
HFO radical scavenging rate and ferric reducing power ((**A**) DPPH scavenging rate, (**B**) OH scavenging rate, (**C**) ABTS scavenging rate, and (**D**) Iron reducing power).

The DPPH radical scavenging activity of HFO was investigated (Figure 2A), and when the concentration of HFO was 5 mg/mL, both HFO variants displayed robust scavenging effects, exceeding 75%. EHFO exhibited a 54.21% scavenging rate at 2 mg/mL, in contrast to the 32.06% scavenging rate of FHFO, highlighting the lower scavenging ability of FHFO at lower concentrations. As the concentration increased to 5 mg/mL, the difference in scavenging ability decreased, indicating an overall increase in the DPPH radical scavenging capacity of HFO.

The OH radical scavenging activity of HFO is shown in Figure 2B, where closely overlapping curves suggest a minimal difference in scavenging ability between the two variants. At a concentration of 4 mg/mL, the HFOs exhibited an OH scavenging activity of approximately 70%, signifying their robust ability to scavenge OH radicals. The ABTS radical scavenging activity of the HFOs indicated close to 70% scavenging activity at high concentrations for both the EHFOs and FHFOs (Figure 2C). Compared with FHFOs, EHFOs displayed a stronger ABTS scavenging ability, achieving 42.85% at 1 mg/mL.

The reducing power of HFO is shown in Figure 2D. FHFO showed lower reducing power than EHFO at 1 mg/mL, but as concentration increased, FHFO exhibited stronger reducing power, surpassing EHFO at higher concentrations. 

### 3.4. Effect of HFO on Liver Index

**Figure 3:**
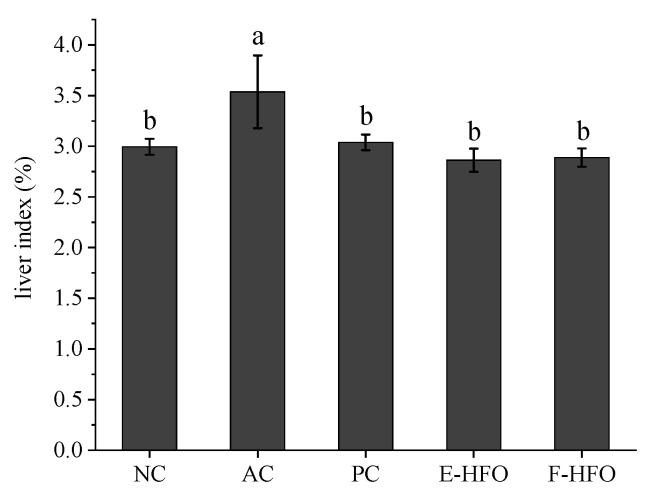
Liver indices of rats. Different letters indicate significant differences. (*p* < 0.05).

The liver index of a rat serves as an indicator of its liver damage [28]. The changes in liver indices of the rats during the experiment are shown in Figure 3. Compared to those in the NC group, rats in the AC group exhibited a significantly elevated liver index (*p* < 0.05), indicating that alcohol-induced toxicity occurred and confirming the success of the modeling process. In contrast to the AC group, both the PC control and HFO intervention groups demonstrated a significant suppression of the alcohol-induced increase in the liver indices, suggesting that HFO has the potential to mitigate alcohol-induced liver injury. 

### 3.5. Effect of HFO on Serum Biochemical Factors

**Figure 4:**
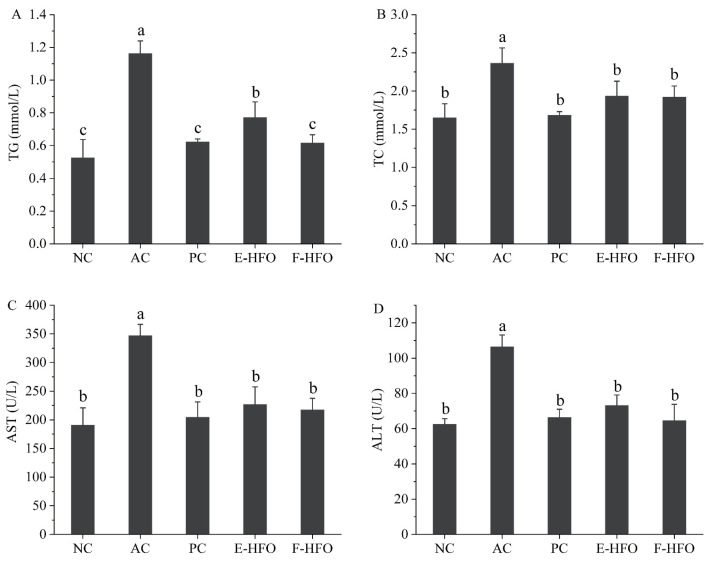
Effect of HFO on serum biochemical indices in rats with alcoholic liver injury ((**A**) TG, (**B**) TC, (**C**) AST, and (**D**) ALT). Different letters indicate significant differences (*p* < 0.05).

Serum levels of TC and TG have been shown to be positively correlated with lipid oxidation [13]. AST and ALT are the main metabolic enzymes in liver cells and can be used as the main sensitive indicators of liver health [14]. These enzymes play a crucial role in assessing liver function. As shown in Figure 4, the levels of serum TC, TG, AST, and ALT were significantly greater in the AC group compared with NC (*p* < 0.05), indicating the successful establishment of the rat model of alcohol-induced liver injury. Alcohol intake resulted in elevated levels of serum TC, TG, AST, and ALT in rats, disrupting lipid regulation. The concentrations of TC, TG, AST, and ALT were markedly reduced in both the PC and HFO groups in comparison to the AC group (*p* < 0.05). Nevertheless, no notable differences were observed when compared to the NC group (*p* < 0.05). These findings indicate that HFO intervention can significantly reduce the serum TC, TG, AST, and ALT levels and improve the negative effects of excessive alcohol intake on the liver.

### 3.6. Effect of HFO on Liver Antioxidant Capacity

**Figure 5:**
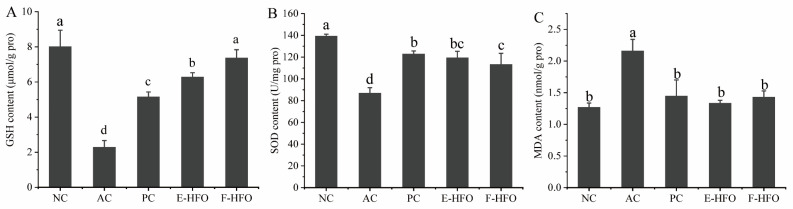
Effects of high Fischer ratio oligopeptides on oxidative stress in the liver of rats with alcohol-induced liver injury ((**A**) effect of HFO on GSH, (**B**) effect of HFO on SOD, and (**C**) effect of HFO on MDA). There were significant differences between the values marked with different letters (*p* < 0.05).

Alcoholic liver injury is associated with dysregulation of hepatic redox homeostasis, and GSH, SOD, and MDA are important indicators of hepatic oxidative status. Figure 5 illustrates that in comparison to the NC group, GSH and SOD levels were significantly lower, and the MDA levels were significantly higher in the AC group. This suggests that alcohol intake led to more pronounced liver injury in rats, confirming the success of the modeling process. The PC group, as well as the E-HFO and F-HFO intervention groups, all demonstrated varying degrees of improvement in rat liver injury. Compared to the model group, the liver GSH and SOD levels in the PC, E-HFO, and F-HFO groups exhibited significant increases, while MDA levels demonstrated significant decreases (*p* < 0.05). Notably, the GSH level in the F-HFO group was significantly higher than that in the E-HFO group at the same dose, and there was no significant difference in the increase in the MDA level and the decrease in the SOD level when compared with the E-HFO group at the same dose (*p* < 0.05). The results indicate that HFO prepared by enzymatic digestion and fermentation has the potential for reducing the level of hydrogen peroxide and alleviating alcohol-induced oxidative stress in the liver.

### 3.7. Effect of HFO on Liver Tissue Status

**Figure 6:**
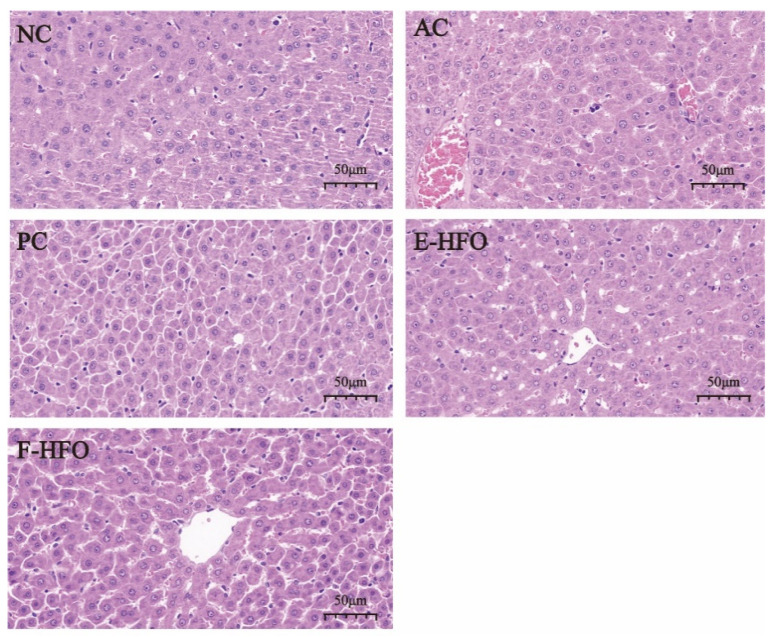
Effect of HFO on the histopathology of the liver of rats with alcoholic liver injury.

The staining of pathological histological sections of the liver from rats with alcoholic liver injury is shown in Figure 6. Hepatocytes exhibited a clear structure, orderly arrangement, normal morphology, uniform size, no foamy variation, and no congestion in the NC group. The nuclei were large and round, with minimal necrosis. In contrast, the boundaries of the liver lobules in the AC group became blurred, the cell cords were disorganized, the liver sinusoids were narrowed, and congestion was evident. The cells exhibited cloudy swelling, foamy or balloon-like variation, and lightly stained cytoplasm, suggesting significant damage to hepatocyte structure due to alcohol. Hepatocyte necrosis and nuclear consolidation were observed, confirming the success of the model. In contrast, the three intervention treatment groups—the PC group, the EHFO group, and the FHFO group—attenuated liver damage to varying extents. The protective effects of the EHFO and FHFO intervention groups were comparable to those of the PC group. Hepatocyte congestion and arrangement showed significant improvement, and the boundaries of hepatic lobules were more clearly defined with no apparent cell necrosis.

### 3.8. Effects of HFOs on the Expression of ADH4, ALDH2, and CYP2E1 in Rat Liver

**Figure 7:**
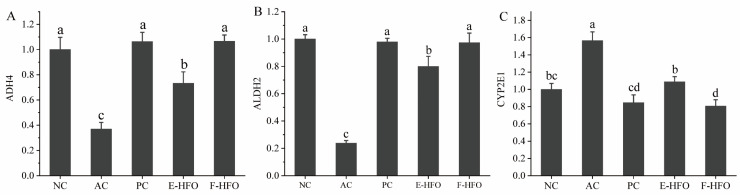
The mRNA expression levels of alcohol metabolism enzymes ((**A**) qPCR analysis of the mRNA level of ADH4, (**B**) ALDH2, and (**C**) CYP2E1 in liver tissue). Different letters indicate significant differences (*p* < 0.05).

The effects of HFO on the mRNA expression levels of alcohol-metabolizing enzymes in the rat liver are shown in Figure 7. Compared with the blank group, the mRNA levels of CYP2E1 in the model group were significantly elevated (*p* < 0.05), while the mRNA expression levels of the related enzymes involved in alcohol metabolism, ADH4 and ALDH2, were significantly reduced (*p* < 0.05). After the metadoxine intervention, the mRNA expression levels of ADH4, ALDH2, and CYP2E1 were not significantly different from those in the normal group, while the effect of EHFO, although slightly weaker than that of FHFO, was still significantly different from that of the model group.

## 4. Discussion

The liver performs crucial functions in nutrient metabolism, oxidative homeostasis, immune regulation, and the maintenance of normal signaling pathways [29]. Excessive alcohol consumption results in severe liver damage, contributing to the emergence of alcoholic liver damage as a global health concern [30]. Alcohol-induced liver damage involves oxidative stress and proinflammatory cytokines [11]. Previous studies have validated the ability of HFO to decrease the levels of MDA, a lipid peroxidation byproduct, while augmenting the levels of SOD and GSH, pivotal antioxidant enzymes in the body [31]. Determining liver injury extent involves assessing serum ALT and AST levels [32], where elevated levels of TC and TG strongly correlate with steatosis [33].

For the first time, the antioxidant activity of the prepared gluten-high Fischer ratio oligopeptides was characterized and analyzed in vitro, and the results showed that HFO had strong DPPH, OH, and ABTS scavenging activities and iron reducing ability in a concentration-dependent manner. Amino acid analysis revealed that the EHFO and FHFO values were 23.436 and 25.195, respectively, in which the BCAA content accounted for 22.092% and 27.721%, respectively, of the total amino acids. The antioxidant capacity of oligopeptides characterized by a high Fischer ratio is presumed to be linked to elevated levels of BCAAs. Previous studies have demonstrated the ability of BCAAs to counteract or mitigate oxidative damage induced by free radicals [34].

Metadoxine accelerates the excretion of ethanol and its metabolites, acetaldehyde and ketone bodies, via the kidneys, thus speeding up the restoration of liver function, protecting liver cell membranes, and preventing liver toxicity caused by excessive alcohol intake [19]. In addition, some studies have shown that metadoxine promotes hepatic glucose synthesis and lipid peroxidation in hepatocytes, which can reduce the inflammation of hepatocytes and the accumulation of fat in alcoholic animals [35]. Therefore, this study used metadoxine as a positive drug to observe the effect of HFO in a controlled manner.

Excessive alcohol intake results in aberrant oxidative stress in the liver. GSH can activate diverse metabolic enzymes, participating in the metabolism of reactive oxygen radicals, and thus plays a key role in protecting cells from oxidative damage as well as in regulating redox homeostasis [36]. The GSH levels in the EHFO and FHFO intervention groups in this study did not significantly differ from those in the blank group, implying its involvement in regulating the metabolism of reactive oxygen radicals. If excessive alcohol intake is not promptly metabolized oxidatively, this results in the production of the lipid peroxidation product MDA, whose content can indirectly indicate the extent of free radical damage to the body [37]. SOD, a crucial enzyme for scavenging oxygen free radicals in cells, can mitigate the damage inflicted by a substantial quantity of oxygen free radicals, thereby fulfilling a protective and reparative role in cells [38]. The experimental findings revealed a significant increase in MDA and SOD levels in the livers of rats with liver injury following the EHFO and FHFO interventions compared to the model group, indicating an improvement in hepatic oxidative stress.

ALDH2 in the mitochondria of liver cells is a crucial alcohol-metabolizing enzyme with substantial activity, capable of eliminating acetaldehyde, an intermediate product in alcohol metabolism associated with cancer and inflammation. Ethanol is extensively oxidized to acetaldehyde by ADH4 after ingestion, and then ALDH2 metabolizes acetaldehyde to acetic acid [39]. Meanwhile, the CYP2E1 metabolic pathway of ethanol amplifies the generation of ROS, such as hydroxyethyl radicals, superoxide anion, and hydroxyl radicals, contributing to oxidative stress [40]. Following HFO intervention with positive drugs, mRNA expression levels of ADH4, ALDH2, and CYP2E1 did not significantly differ from the blank group. However, EHFO, while slightly less effective than FHFO, still exhibited a significant difference from the model group. These findings suggested that supplementing with HFO effectively enhances the liver alcohol metabolism by decreasing CYP2E1 mRNA expression and increasing ADH4 and ALDH2 mRNA expression, thereby ameliorating liver injury. In this study, we systematically investigated the protective effects of HFO, which was prepared using both enzymatic and fermentation methods, against alcoholic liver injury. Our results illustrate that HFO effectively mitigated alcohol-induced liver injury in rats by modulating lipid metabolism and elevating mRNA expression levels of enzymes related to ethanol metabolism, consequently alleviating oxidative stress. HFO can be produced through enzymatic hydrolysis or biofermentation. The biofermentation method, as a novel approach, offers advantages of environmental friendliness, cost-effectiveness, and simplicity. Moreover, compared with HFO prepared via enzymatic hydrolysis, HFO prepared via biofermentation may exhibit greater and more diverse biological activities.

## 5. Conclusions

A model of alcohol-induced liver injury was successfully established in this study. HFO intervention can ameliorate alcohol-induced liver injury in rats by reducing dyslipidemia, mitigating oxidative stress damage, and increasing the mRNA expression levels of alcohol-metabolizing enzymes. The hepatoprotective mechanism of HFO may be linked to the upregulation of alcohol dehydrogenase, an enzyme involved in alcohol metabolism. Furthermore, HFO can mitigate oxidative stress by increasing the levels of hepatic SOD and GSH. Additionally, HFO regulates lipid metabolism by reducing serum TG and TC levels, thus improving pathological changes in the liver caused by lipid accumulation. For pharmaceutical applications, we recommend an initial intake dosage of 50 mg/kg/day. These findings propose HFO as a promising therapeutic agent for preventing alcoholic liver injury. Consequently, this study offers valuable insights and methodologies for the development of functional active proteins derived from plants, serving as a valuable resource for the pharmaceutical and food industries.

## Figures and Tables

**Table 1:** The nucleotide sequence of the PCR primers for amplification.

Gene	F Primer	R Primer
ADH4	GGGAACGTTCTTTGGTGGTTG	ATCAGGTCAATCGCGTCGTT
ALDH2	ATCCTCGGCTACATCAAATCG	GTCTTTTACGTCCCCGAACAC
CYP2E1	CAAGTCTTTCACCAAGTTGGCA	CCCCCGTCCAGAAAACTCAT
GAPDH	GGTGAAGGTCGGTGTGAACG	CTCGCTCCTGGAAGATGGTG

**Table 2:** Amino acid composition analysis of HFO.

Free Amino Acids	EHFO	FHFO	Type
Asp	1.168	0.657	
Thr	0.939	0.634	
Ser	1.696	0.782	
Glu	10.611	7.435	
Gly	1.176	1.000	
Ala	1.032	1.526	
Cys	0.175	0.126	
Val	2.380	2.320	BCAAs
Met	0.465	0.548	
Ile	1.868	1.801	BCAAs
Leu	2.224	2.722	BCAAs
Tyr	0.128	0.127	AAAs
Phe	0.149	0.145	AAAs
His	0.796	0.883	
Lys	0.576	0.601	
Arg	0.981	0.432	
Pro	2.933	2.946	

**Table 3:** The Fischer ratio of EHFO and FHFO.

Free Amino Acids	EHFO	FHFO
BCAAs	6.472	6.843
AAAs	0.276	0.272
Fischer ratio	23.436	25.195

BCAAs: branched-chain amino acid; AAAs: aromatic amino acids; Fischer ratio = (Val + Ile + Leu)/(Tyr + Phe).

## Data Availability

The original contributions presented in the study are included in the article, further inquiries can be directed to the corresponding authors.
